# Supplementary material for: Reoperation for Recurrent and Persistent Cushing’s Disease without Visible MRI Findings
Source: J Clin Med. 2022 Nov 20;11(22):6848. doi: 10.3390/jcm11226848 (PMC9699622; doi:10.3390/jcm11226848)
Supplement: Supplementary file 1 [file jcm-11-06848-s001.zip › jcm-1987098-supplementary.pdf]

**Table S1.** Preoperative and postoperative evaluation of the repeated surgery of 42 patients

| Patient No. | Gender/ Age (yrs) | 1st Pathology | MRI | HDDST | BIPSS | Interval between $1^{st}$&$2^{nd}$ surgery (months) | Remission | 2nd Pathology | 2nd Post-OP Follow-up (months) |
|---|---|---|---|---|---|---|---|---|---|
| Recurrent CD | | | | | | | | | |
| #1 | F/66 | NA | + | + | + | 216 | R | PA- | 13 |
| #2 | F/74 | NA | + | + | + | 199 | R | PA+ | 48 |
| #3 | F/70 | PA | + | - | NP | 195 | R | PA+ | 1 |
| #4 | F/52 | NA | - | + | + | 151 | R | PA+ | 38 |
| #5 | M/30 | PA | - | + | + | 90 | R | PA+ | 147 |
| #6 | F/61 | PA+ | + | + | NP | 116 | R | PA+ | 82 |
| #7 | M/23 | PA+ | - | + | NP | 25 | N | PA+ | 26 |
| #8 | F/54 | PA | - | + | + | 103 | R | PA+ | 46 |
| #9 | F/49 | PA+ | - | + | NP | 87 | N | - | 68 |
| #10 | F/48 | NA | + | + | NP | 112 | R | PA+ | 43 |
| #11 | F/57 | PA+ | - | + | NP | 69 | R | PA | 64 |
| #12 | F/40 | PA- | + | + | + | 91 | N | - | 45 |
| #13 | M/35 | PA+ | + | + | + | 32 | R | NA | 1 |
| #14 | F/45 | PA+ | + | + | NP | 57 | R | PA+ | 74 |
| #15 | F/61 | PA- | + | + | NP | 87 | R | PA+ | 1 |
| #16 | F/25 | PA- | - | - | + | 24 | R | PA+ | 91 |
| #17 | F/70 | hyperplasia | + | + | + | 7 | R | - | 107 |
| #18 | F/55 | PA+ | - | + | NP | 14 | R | PA+ | 32 |
| #19 | F/38 | PA+ | + | + | NP | 18 | R | - | 7 |
| #20 | F/33 | PA+ | + | + | NP | 39 | N | hyperplasia | 54 |
| #21 | F/34 | PA+ | - | NP | NP | 10 | N | - | 29 |
| #22 | F/34 | PA+ | + | + | NP | 65 | N | - | 6 |
| #23 | F/48 | PA+ | + | + | NP | 75 | R | PA+ | 5 |
| #24 | F/46 | PA+ | + | + | + | 92 | R | - | 15 |
| #25 | F/66 | PA+ | + | + | NP | 48 | R | PA+ | 1 |
| #26 | F/54 | PA+ | + | + | NP | 168 | R | PA+ | 1 |
| #27 | F/38 | - | + | + | + | 50 | R | PA+ | 17 |
| Persisting CD | | | | | | | | | |
| #28 | M/28 | PA+ | + | + | NP | 37 | R | PA- | 4 |
| #29 | M/33 | NA | - | - | + | 43 | R | PA+ | 1 |
| #30 | F/45 | PA- | + | - | NP | 37 | N | PA+ | 32 |
| #31 | M/41 | NA | + | + | + | 61 | R | - | 73 |
| #32 | M/18 | NA | + | + | NP | 1 | R | PA+ | 1 |
| #33 | F/45 | PA+ | - | + | NP | 20 | N | PA+ | 2 |
| #34 | M/28 | PA+ | - | + | NP | 6 | N | PA- | 151 |
| #35 | F/29 | PA- | + | + | + | 43 | N | - | 4 |
| #36 | F/46 | NA | + | + | + | 6 | R | PA+ | 6 |
| #37 | F/35 | PA+ | + | + | NP | 5 | R | PA+ | 35 |
| #38 | F/19 | PA+ | + | + | NP | 8 | N | PA+ | 12 |
| #39 | F/51 | PA+ | + | + | NP | 7 | N | PA+ | 16 |
| #40 | M/45 | PA | - | + | - | 24 | N | - | 4 |

| | | | | | | | | | |
|---|---|---|---|---|---|---|---|---|---|
| #41 | F/22 | PA+ | + | + | + | 4 | R | PA+ | 5 |
| #42 | F/39 | - | + | + | + | 3 | R | - | 3 |

Age, age at 2st operation; Pathology PA+, ACTH-positive staining pituitary adenoma; Pathology PA-, ACTH-negative staining pituitary adenoma; Pathology PA, pituitary adenoma without available immumohistochemical staining result; Pathology -, no pathological evidence of pituitary adenoma; Pathology NA, no available pathological record; MRI +, adenoma identified, MRI -, no adenoma identified; LDDST/HDDST -/+, suppression of both serum cortisol and 24h urinal free cortisol after high-dose dexamethasone but no after lose-dose; HDDST -/-, failure of suppression after either high-dose or low-dose dexamethasone; BIPSS +, central to peripheral ACTH gradients >=2 without CRH/vasopressin stimulation; BIPSS -, central to peripheral ACTH gradients <2 without CRH/vasopressin stimulation; Remission R, remission; Remission N, not remission; NP, not performed.
